# Genomic vulnerability assessment reveals the potential benefits of adaptive introgression by mitigating the maladaptive risk of admixed populations

**DOI:** 10.48130/forres-0025-0026

**Published:** 2025-11-19

**Authors:** Wen-Hao Li, Han-Yang Lin, Chen Chen, Chen-Feng Lin, Xing-Xing Shen, Yun-Peng Zhao

**Affiliations:** 1 State Key Laboratory for Vegetation Structure, Function and Construction (VegLab), MOE Key Laboratory of Biosystem Homeostasis and Protection, College of Life Sciences, Zhejiang University, Hangzhou 310058, China; 2 Zhejiang Provincial Key Laboratory of Plant Evolutionary Ecology and Conservation, School of Life Sciences, Taizhou University, Taizhou 318000, China; 3 College of Agriculture and Biotechnology, Zhejiang University, Hangzhou 310058, China; 4 Centre for Evolutionary & Organismal Biology, Zhejiang University, Hangzhou 310058, China

**Keywords:** Climate change, Genomic offset, Intraspecific introgression, Gradient forest, Davidia involucrata

## Abstract

As climate change accelerates, plant species largely rely on genetic variation to adapt and survive when they fail to track their ecological niches through range shifts. Predicted genomic vulnerability is able to identify populations lacking the necessary genetic variation for climate change adaptation. However, the role of introgression in genomic vulnerability remains poorly explored. Here, we used the dove tree (*Davidia involucrata*), a relict species native to southwestern China, to test whether introgression may reduce genomic vulnerability. By integrating population genomics and environmental data collected from 196 individuals of 18 populations, we identified 747 strictly climate-associated loci across the distribution range of *D. involucrata*, 138 of which were recovered from the genetically admixed populations. We estimated the genomic vulnerability for three genetic lineages and two admixed groups using the gradient forest approach, and found that eastern populations are likely to be at higher risk. The eastern admixed populations exhibited a significant reduction, with introgression from the southern lineage. Cumulative importance analysis showed moderate importance for introgressive loci along environmental gradients. This indicates that the introduction of novel alleles through introgression provides only a partial and insufficient counterbalance to the maladaptation observed in *D. involucrata* under climate change. Our study highlights the role of intraspecific introgression in response to climate change and emphasizes the importance of genomic vulnerability studies in informing conservation practices for relict and endangered species.

## Introduction

Many species are increasingly faced with a higher risk of maladaptation caused by rapid climate change^[1,2]^. To escape demographic decline or extinction, species respond by tracking their ecological niche, persisting in the local habitat through phenotypic plasticity, or genetic adaptation to new conditions^[3,4]^. However, range shifting may be difficult for many species^[5]^, especially for sessile organisms like plants. Therefore, exploring the evolutionary potential of species for future adaptation is not only crucial to understanding whether and how organisms can persist in the context of climate change but can also benefit conservation and management strategies to cope with global biodiversity loss^[4,6]^.

Predictions of genomic vulnerability are increasingly used to assess the extent of genomic change required for populations to track climate change through evolutionary adaptation^[4,7−9]^. These methods integrate genetic, phenotypic, and ecological data to identify the adaptive loci underlying local adaptation^[9]^ and then predict the disruption between genotype and the future environment by using associations across current gradients as a baseline^[7,8]^. Populations with greater mismatches (i.e., genomic offsets) between their current genetic composition and future climate conditions are expected to be more vulnerable to climate change. Although many studies have considered the contributions of migration and adaptation at the population level to environmental changes in predictions of genomic vulnerability, less attention has been given to other evolutionary processes, such as introgression, which can also influence species' responses to climate change^[10,11]^.

Introgressive hybridization (or introgression) is the permanent incorporation of genes from one population into the genome of another reproductively integrated population through a series of crossing and backcrossing events^[12]^. This evolutionary process can either promote or hinder local adaptation^[13]^. Gene flow or introgression from better-adapted populations (or species) could enrich gene pools and provide important adaptive alleles^[14]^. An increasing number of studies have shown that gene flow can promote adaptation, and that local adaptations can be maintained despite high gene flow^[15]^. In order to mitigate the maladaptation of populations that lack adaptive variations, assisted gene flow is regarded as a powerful tool for conservation management^[16]^. However, such options are controversial, involving the risks of swamping and disrupting coadapted gene complexes in local gene pools, potentially leading to outbreeding depression^[13]^. Recent research has shown that natural hybridization from warm-adapted species can reduce the genomic vulnerability of several narrowly distributed species by enhancing their adaptive potential^[17]^. These results provided evidence of gene flow mitigating maladaptation, but these preliminary conclusions may be case-specific; therefore, more studies are required^[18]^.

Here, we assessed the influence of introgression events on genomic vulnerability in a tertiary relict plant, the dove tree (*Davidia involucrata* Baill.). It is a rare and endangered tree species. Most of the investigated populations of *D. involucrata* have small population sizes and extremely low genetic diversity, restricted to the humid mountainous regions in south-central and southwestern China^[19]^. However, the genetic differentiation across its range is relatively strong^[20]^. Analyses using different genetic markers have revealed a significant east–west divergence among *D. involucrata* populations, driven by geographical factors^[20−22]^. A recent research based on genome-resequencing data discovered a finer population structure, where the previously claimed western lineage was further divided into two lineages, and recurrent ancestral hybridizations were determined^[22]^. These admixed populations serve as ideal materials for investigating genetic response and adaptive potential under climate change, further providing a great opportunity to assess the consequences of introgression for genomic vulnerability.

Here, we performed restriction-associated DNA sequencing (RAD-seq) of 186 individuals from 16 populations and retrieved the published resequencing data for 10 individuals from two populations across the natural distribution range of *D. involucrata* in China. We first inferred the formation of admixed populations of *D. involucrata*. We then identified the single nucleotide polymorphisms (SNPs) potentially involved with local adaptation through genome–environment association (GEA) analysis. Finally, we predicted the integrated genomic offsets by incorporating the impacts of local adaptation and artificial migration on the fate of populations under future climate scenarios. We aimed to address the following questions: (1) How did evolutionary processes contribute to the introgression in *D. involucrata*? (2) How would *D. involucrata* populations respond to climate change? (3) Did introgression alleviate the genomic vulnerability of admixed populations in *D. involucrata*?

## Materials and methods

### Sampling and sequencing

**Figure 1:**
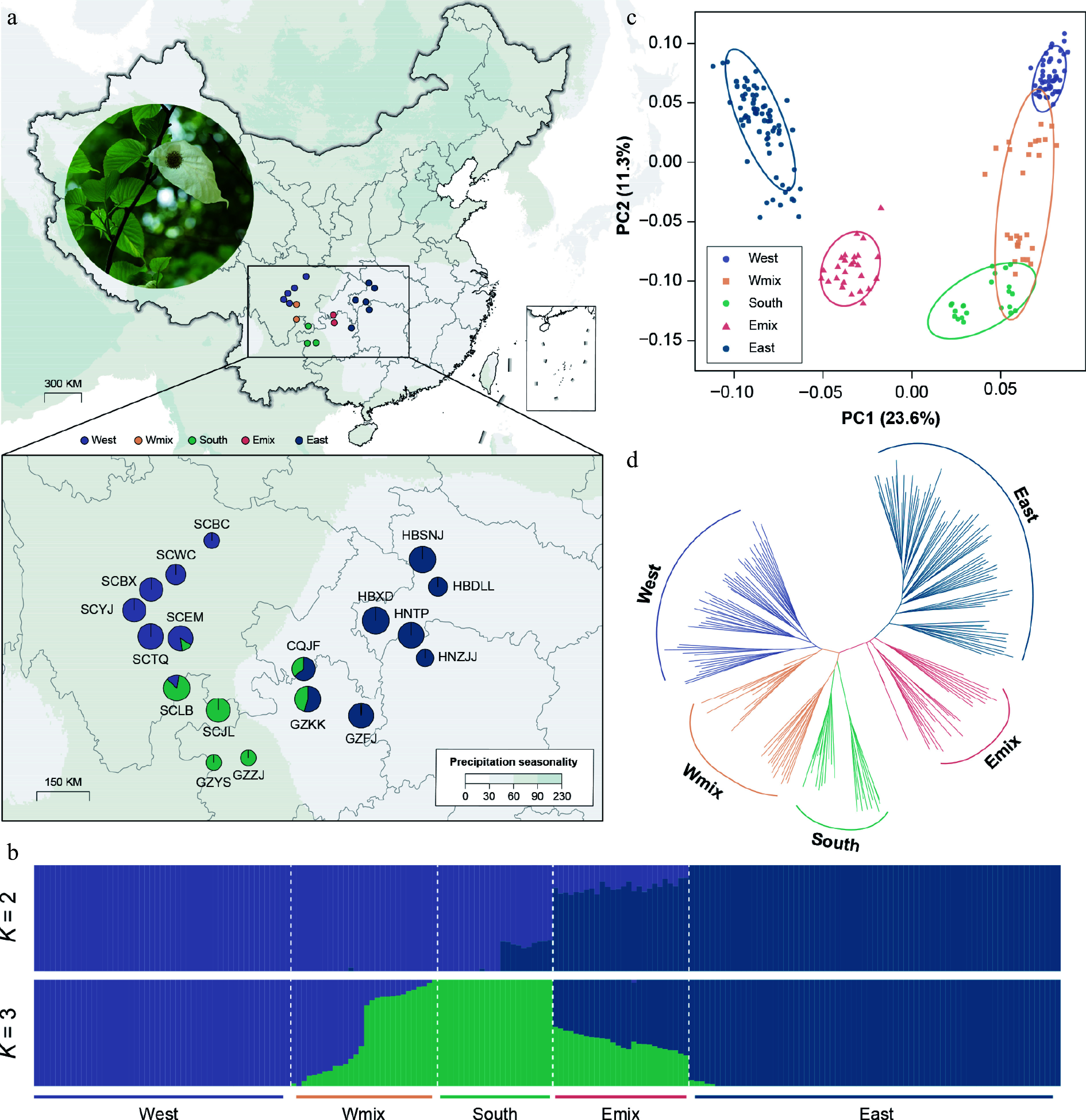
Genetic structure, variation, and differentiation of *Davidia involucrata*. (a) Geographic distribution of 18 natural populations, with a photo of the dove tree. Points with colors represent individual ancestries inferred by the admixture analysis (*K* = 3). (b) Population assignment using ADMIXTURE with *K* = 2 and *K* = 3. The height of each colored segment represents the proportion of the individual ancestries. (c) Principal component analysis (PCA), showing five genetic groups of *D. involucrata*. (d) The NJ tree of *D. involucrata*.

We sampled a total of 196 individuals from 18 populations of *D. involucrata* along a broad environmental gradient around the Sichuan Basin, China (Fig. 1a; Supplementary Table S1), from which 186 individuals from 16 populations were collected by the authors. Considering the absence of the southern lineage identified by Ren et al.^[22]^, we downloaded the resequencing data of 10 individuals from two populations (GZYS and GZZJ) from Guizhou province. This collection covers the major phylogeographic lineages and admixed groups identified in previous studies^[20−22]^.

A modified cetyltrimethylammonium bromide (CTAB) protocol was used to extract genomic DNA from dried leaf materials. DNA samples were subject to RAD-seq to obtain genome-wide SNP markers. The genomic DNA was digested with the *Mse*I-*Taq*I restriction enzyme pair. Next, the DNA libraries were prepared, and Illumina HiSeq 2500 instruments were used to generate 150-bp paired-end reads (with a 400-bp insert size) at Majorbio (Shanghai Majorbio Bio-pharm Technology Co., Ltd.).

### Single nucleotide polymorphism calling and filtering

For data processing, 186 RAD-seq data and 10 resequencing data samples were subject to the same pipeline. We used the process_radtags tool from STACKS v. 2.2^[23]^ to demultiplex and clean the data. Trimmomatic v. 0.39^[24]^ was used to process the raw reads by removing adapters and filtering low-quality reads (*Q* < 20; the proportion of N bases > 10%). Using BWA v. 0.7.17^[25]^, the filtered reads were aligned to the reference genome of *Davidia involucrata* (The National Genomics Data Center, PRJCA001721)^[26]^. Sequence alignment/map (SAM) format files were fed to SAMTOOLS v. 1.2^[27]^ for sorting and mapping the rate statistics (Supplementary Table S2). PICARD v. 1.92 (https://github.com/broadinstitute/picard/) was used to remove polymerase chain reaction (PCR) duplicates.

We applied bcftools v1.5 "mpileup" for calling potential variants for RAD-seq data and resequencing data separately^[28]^. For RAD-seq data, we excluded low-quality variants on the basis of the following annotation values: Quality score < 30.0; quality by depth (QD) < 5.0; genotype read depth (DP) < 4 and DP > 150; strand odds ratio (SOR) < 10.0. For resequencing data, both monomorphic and polymorphic variants were kept. We also excluded low-quality variants with same criteria, except for a different DP threshold: DP < 70 and DP > 630 (one-third to threefold the average depth). We then merged the RAD-seq dataset and resequencing dataset. In addition, we used VCFtools v. 0.1.15^[29]^ to retain only diallelic SNPs with < 40% missing data, and minor allele frequency (MAF) ≥ 0.01. We also tested the potential bias of data mixing by comparing the SNPs' frequency (Supplementary Fig. S1).

### Estimation of genetic diversity and population structure

To estimate the genetic diversity of *D. involucrata*, we calculated the average values of *π*, heterozygosity, and pairwise *F*_*ST*_ across all populations by using the "population" module in STACKS. We used the coverage size (~6,973 kb) of RAD-seq data to correct the estimation of *π*. Meanwhile, we removed SNPs subject to strong linkage disequilibrium (LD) (*r*^2^ > 0.2) for the population structure and demographic analyses using PLINK v. 1.90b4.9^[30]^ (--indep-pairwise 100 10 0.2). This resulted in a dataset of 4,170 LD-pruned SNPs. The structure and admixture of the studied populations were inferred using ADMIXTURE v. 1.30^[31]^, with five replicates and 10-fold cross-validation (CV) from *K* = 2 to 10. The best *K* value was determined by the lowest CV value. Principal component analysis (PCA) was conducted to determine the genetic relatedness among samples using PLINK (-pca). For phylogenetic analysis, we generated a neighbor-joining (NJ) tree using MEGA X.

### Inference of demographic history and gene flow

We reconstructed the demographic history of the effective population size (*Ne*) over time using the pairwise sequential Markovian coalescence (PSMC) method^[32]^. On the basis of the population structure, we divided all populations into five groups, including three pure lineages (*Q* > 0.999 in the admixture analysis when *K* = 3) and two admixed groups. For each population group, we downloaded the resequencing data of representative individuals (YJ17, YL14, YS6, KKS1, and SNJ5) from Ren et al.^[22]^ for the PSMC analysis, assuming a mutation rate of 1.87 × 10^−9 [26]^ and a 10-year generation time. On the basis of 4,170 neutral SNPs, we generated folded site frequency spectra (SFSs) for each group with easySFS^[33]^. These SFSs were used as input for StairwayPlot v. 2.1, which effectively reflects the *Ne* dynamic in recent periods^[34]^.

To reveal the demographics of *D. involucrata* populations, we carried out coalescent simulations with fastsimcoal v. 2.7.0.6^[35]^ based on the composite likelihood method. On the basis of the reported demographic history of the three lineages^[22]^, we incorporated two admixed groups and developed eight alternative models involving the five groups. These models hypothesized no gene flow, only ancient gene flow, and gene flow among all population group pairs. We also tested whether migration events occurred in the admixed populations (Supplementary Fig. S2). To ensure convergence, the program was run 40 times for each model. Estimates were generated from 100,000 simulations per likelihood estimation (-n100,000 to N100,000), 40 expectation-conditional maximization (ECM) cycles (-L40), and 50 runs for each dataset. Akaike's information criterion (AIC) was applied to compare the quality of the sets of statistical models with each other and determine the best one (Supplementary Table S3). The generation time and the mutation rate were the same as above. Finally, 95% confidence intervals were calculated from the 100 datasets of the best model (Supplementary Table S4).

Additionally, we used *Dsuite* v. 0.5 to assess gene flow between admixed and pure lineages^[36]^. Patterson's *D* statistics were summarized using the *Dtrios* function, based on the assumed topology (((P1,P2),P3),O). In the absence of a true outgroup, we set two assumed topologies based on the NJ tree. Specifically, we selected populations with the highest pairwise *F*_*ST*_ as pseudo-outgroups. When we tested for the genetic admixture of the western and southern lineages (the Wmix group, hereafter; see the results), we assigned the eastern lineage (HBZJJ) as the outgroup and detected introgression in western–Wmix–southern trios. When we tested for the genetic admixture of the eastern and southern lineages (the Emix group, hereafter), we considered the western lineage (SCBC) as the outgroup and detected introgression in eastern–Emix–southern trios. The trios with a nonzero *D-*statistic and |*Z*-score| > 3 were regarded as significant introgression signals between P2 and P3^[37]^. We also calculated the introgressive region using *Dinvestigate* function for two admixture groups; default parameters (-w 50, 25) were used. Windows with positive *D-*statistics and *f-dM* > 0.15 were regarded as the introgression region.

### Climate variables

To characterize the present climatic conditions at the sampling locations of *D. involucrata*, we downloaded the layers of 19 bioclimatic variables at the 2.5 arc-min resolution (1970–2000) from the WorldClim v 2.1 database (www.worldclim.org). To describe future climate conditions, we downloaded the BCC-CSM2-MR model layers, including 19 bioclimatic variables from four shared socioeconomic pathways (SSPs) at the 2.5 arc-min resolution for these time periods: 2041–2060, 2061–2080, and 2081–2100. In addition to the 19 climatic variables available from Worldclim, we included spatial variables. Since a strong isolation × distance effect was detected in *D. involucrata*^[22]^, we added Moran's eigenvector map (MEM) variables as spatial variables^[7]^. We derived the MEM variables from the geographic coordinates of the sampling locations using the R package adespatial v. 0.3-24^[38]^. The top five uncorrelated MEM eigenfunctions were used to model spatial genetic variation.

### Genotype–environment association analysis

We used two methods to identify candidate loci responding to five uncorrelated (Pearson's *r* < 0.7) climatic variables (BIO1, BIO2, BIO7, BIO12, and BIO18) (Supplementary Fig. S3). First, we performed a latent factor mixed model (LFMM) analysis. Ten independent runs with 10^5^ iterations after a 5 × 10^4^ burn-in step were produced to compute the correlations between allele frequencies and climate variables in the R package LEA v. 3.12.2^[39]^. The number of latent factors was set to three according to the results of the inferred population structure (see results). Correlations between SNPs and climatic data were considered significant at *p* < 0.05. Next, we adopted redundancy analysis (RDA) to identify environmental-associated loci with the R package vegan v. 2.6-4^[40]^. We then retained the intersection of the SNP datasets obtained from the two methods for further analysis. Furthermore, we annotated these SNPs (2.5 kb upstream or downstream of the genes) with SNPEff v. 5.2c^[41]^. These protein-coding genes were subsequently annotated by querying against the Swiss-Prot public database, using NCBI BLAST+ v. 2.2.31 with an E-value of 1 × 10^−5^ as the cutoff. We then tested whether these genes were statistically associated with Gene Ontology (GO) terms using the R package topGO v. 2.52^[42]^.

### Mapping range-wide genomic variation and predictions of genomic vulnerability

To map the genomic variation of *D. involucrata* in a geographic sense, we used MAXENT v. 3.4.1^[43]^ with 19 bioclimatic variables to model the current distribution. Collection and filtering of the occurrence datat were carried out via the pipeline described in Tang et al.^[19]^. We computed 20 runs with a regularization multiplier gradient from 0.1 to 2.0 (with a step size of 0.1). Each run took 10 replicates, with maximum iterations of 500 and the convergence threshold of 1 × 10^−5^.

Using the candidate SNPs identified in the GEA analysis described above, we tested which environmental variables best explained adaptive genetic variation by using the gradient forest (GF) model with the R package gradientForest v. 0.1-37^[44]^. Using a set of trees (*n* = 500), the geneome–environment relationship was built for each SNP and then combined into an aggregate function. The GF analysis output the relative predictive power of all environmental variables used. On the basis of the GF model, we transformed 19 climate variables from 12 climate scenarios into genetic importance, and then calculated the Euclidean distance for each cell between the current and future layers^[7]^. To test whether admixed populations showed lower vulnerability, we generated the rough range of each lineage and the admixed population groups. For genetic offsets projected under the SSP245 and SSP585 scenarios (2081–2100), Wilcoxon rank-sum tests were applied to compare the differences among the five groups.

Besides assessing the classic (local) genomic offset associated with the genes that quantify the risk of being maladapted to the future climate *in situ* (without migration), we used two additional metrics considering migration, following Gougherty et al.^[45]^. We predicted the comprehensive vulnerability by calculating three kinds of genomic offset across the current range: the local, the forward, and the reverse offset for the 2081–2100 period under the SSP245 and SSP585 scenarios. To visualize these offsets simultaneously, we stacked the three layers into an red–green–blue (RGB) map, and the specific area was shown in a national range map (Supplementary Fig. S4).

## Results

### Population structure and demographic history

In total, 1.2 Tb of RAD-seq data and 87.4 Gb resequencing data were obtained from 196 samples of 18 populations, with an average data size of 2.59 Gb (Fig. 1a, Supplementary Tables S1 and S2). The average mapping rate of the raw reads to the reference *D. involucrata* genome^[26]^ was 84.8%. After filtering low-quality reads and loci with high missing proportions, we retained 16,569 SNPs (MAF ≥ 0.01) for subsequent analyses.

The clustering analysis revealed a strong population stratification based on 4,170 LD-pruned SNPs. With the lowest CV error in the admixture analysis (Supplementary Table S5), the *K* = 3 scenario suggested that the eastern lineage (HNTP, HNZJJ, HBXD, HBDLL, and HBSNJ) comprised pure individuals from Hunan, Hubei, and Guizhou, whereas individuals from Sichuan belonged to the western lineage (SCWC, SCBX, SCTQ, and SCYJ) and the southern lineage (SCJL, GZZJ, and GZYS) (Fig. 1b). The eastern lineage (π = 2.11 × 10^−4^) showed much higher diversity than the western lineag (π = 1.40 × 10^−4^) (Supplementary Table S1). Meanwhile, we identified 41 hybrid individuals from two hybrid zones, 15 of which showed a genetic admixture of the western and southern lineages (the Wmix group), whereas the other 26 showed a genetic admixture of the eastern and southern lineages (the Emix group). The fixation index suggested a strong population structure between the western and eastern lineages (mean *F*_*ST*_ = 0.35) and between the southern and eastern lineages (mean *F*_*ST*_ = 0.31), as well as between the western and southern lineages (mean *F*_*ST*_ = 0.26). The highest pairwise *F*_*ST*_ value was found between HBZJJ and SCBC (0.52) (Supplementary Fig. S5). The first and the second principal components (PC1 and PC2) explained 23.6% and 11.3% of the total genomic variance, respectively (Fig. 1d). PC1 contrasted individuals from the western lineage and the eastern lineage (Fig. 1c), which is consistent with the *K* = 2 scenario from the admixture analysis. Meanwhile, PC2 indicated the genetic differentiation among the western lineage, the Wmix group, and the southern lineage. The results from the NJ tree also supported the evolutionary affinity and the shallow divergence pattern of these three groups (Fig. 1d).

**Figure 2:**
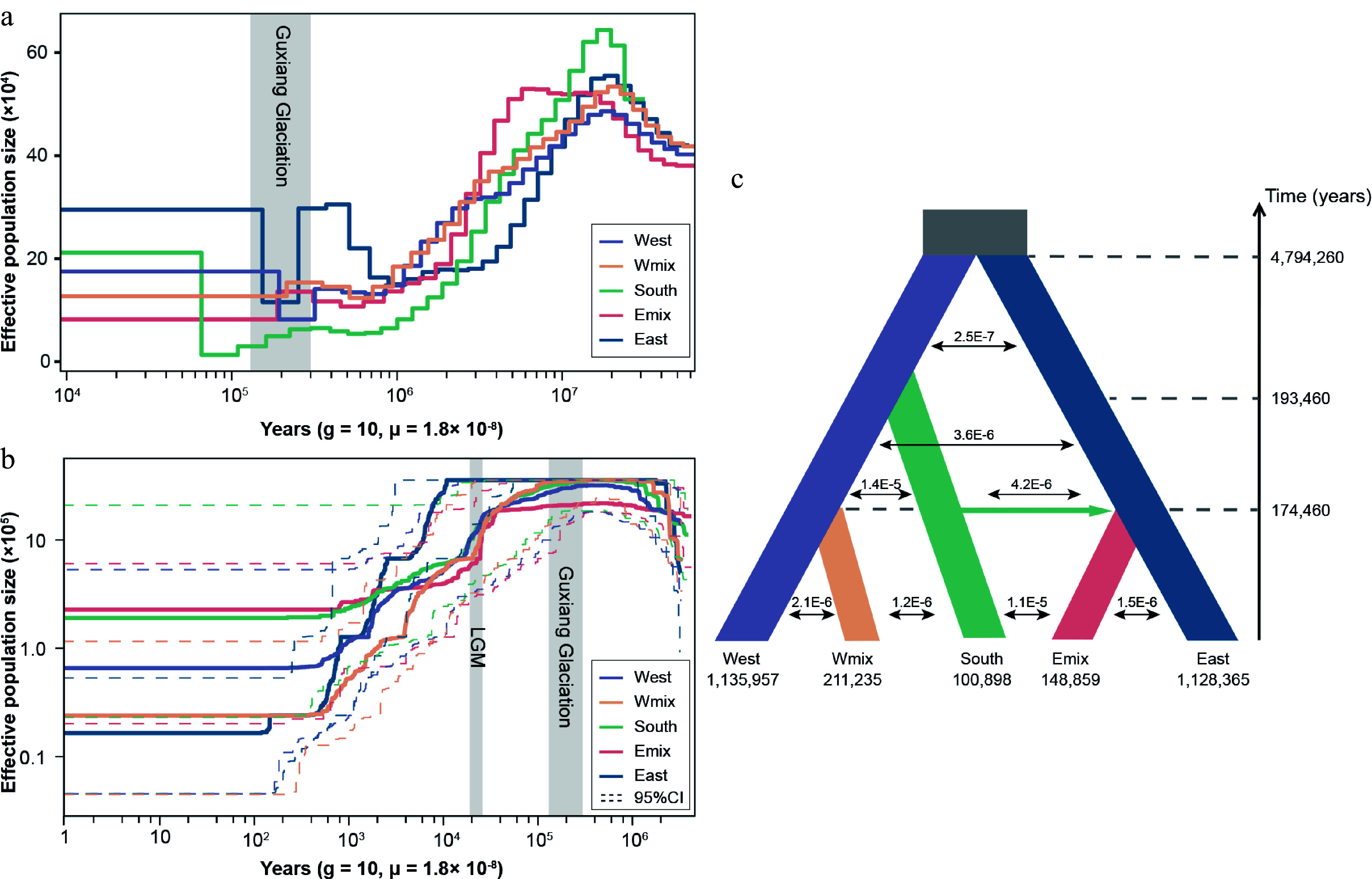
Demographic history of three lineages and two admixed groups in *Davidia involucrata*. (a) Effective population size dynamics inferred by pairwise sequentially Markovian coalescence (PSMC). (b) Effective population size dynamics inferred by StairwayPlot. Dashed lines represent the 95% confidence intervals (c) Divergence times, effective population sizes, and migration rates inferred for *Davidia involucrata* lineages using fastsimcoal2.7. The grey rectangles refer to the Guxiang glaciation periods and the last glacial maximum (LGM).

To understanding the formation of the two admixed populations, we first reconstructed their effective population size by two methods. The results for PSMC showed that the population size experienced a long-term decrease after the peak around 10 million years ago (Mya) (Fig. 2a). Five population groups had a common population expansion around 50–90 thousand years ago (kyr). During the Guxiang glaciation, all populations contracted, but the eastern and western lineages recovered their population size subsequently (Fig. 2a). The simulation based on SFS with stairwayplot showed a similar pattern of a decrease in *Ne*, especially during the last glaciation maximum (LGM), but no recent population expansion was detected (Fig. 2b). We then inferred the demographic history via coalescent simulations (Fig. 2c). Eight models involving five genetic groups were tested in fastsimcoal2.7 (Supplementary Fig. S2). Model 7 exhibited the lowest AIC value and the highest likelihood (Supplementary Table S3). The best model suggested that the ancestor of the western and eastern lineages diverged roughly 4.8 Mya. The split of the Wmix group and the Emix group from the southern lineage dated back to ~0.17 Mya; this time is consistent with the population contraction and re-expansion of the western and eastern lineages. Meanwhile, a migration event occurred from the southern lineage to the Emix group, suggesting a second contact event between the southern and eastern lineages. The level of gene flow between the southern lineage and the Emix population group was higher than that observed between other population pairs (Fig. 2c). At the split time of admixed populations, all populations experienced a sharp contraction in population size (Fig. 2a, b). Subsequently, the levels of gene flow decreased, suggesting reduced connectivity between populations.

The *D* statistics also supported the results of the fastsimcoal analysis (Table 1). We found strong evidence of introgression with a significant excess of ABBA (positive *D*) for the Wmix and the Emix groups. This result indicated two introgression events, one from the southern lineage to the Wmix group and another from the southern lineage to the Emix group.

**Table 1:** Evidence for introgression from pure lineages to admixed populations using *D* statistics calculated by *Dsuite*.

P1	P2	P3	O	D	*Z*-score	f4-ratio	BBAA	ABBA	BABA
West	Wmix	South	East	0.22	9.04	0.101	227.4	160.4	102.1
East	Emix	South	West	0.18	6.12	0.082	514.3	153.2	105.7

### Climate-related candidate genes

**Figure 3:**
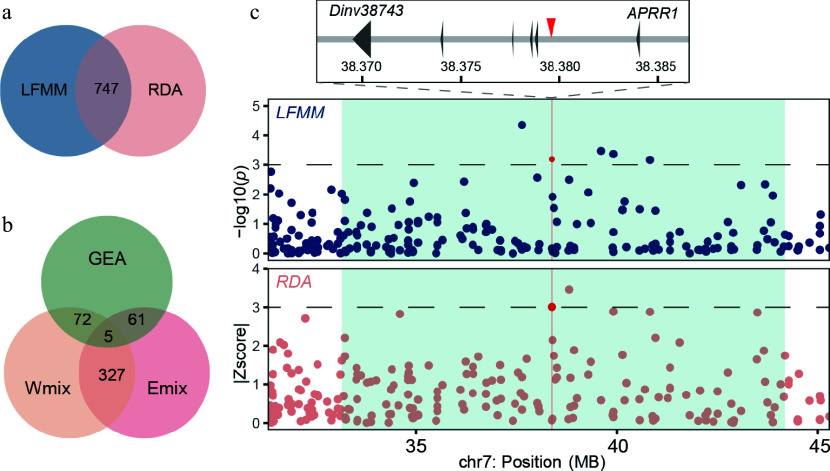
Genome-wide screening of the loci associated with local environmental adaptation. (a) Venn plot of outliers from the LFMM analysis and RDA. (b) Venn plot of alleles from GEA outliers and introgressive area of the Emix and Wmix groups. (c) Manhattan plot for variants associated with the seasonality of temperature (BIO4) in the LFMM analysis (upper panel) and the annual precipitation (BIO12) in the RDA (lower panel). Dashed horizontal lines refer to significance thresholds, and red points indicate the significant alleles (chr7: 38379580) in the introgressive area. The introgression windows are shown as light blue rectangles.

By employing two complementary GEA methods, we identified genetic variants associated with environmental factors across the distribution range of the dove tree. Using LFMM, we tested the GEAs for 19 environmental variables and identified 1,844 SNPs significantly associated with one or more environmental variables (Supplementary Fig. S6). RDA was used to identify covarying allele frequencies associated with the multivariate environment. Five variables (BIO01, BIO02, BIO07, BIO12, and BIO18) with |*r*| < 0.7 were retained for the RDA analyses to avoid multicollinearity (Supplementary Fig. S3). In total, 944 climate-associated SNPs were identified by RDA (Supplementary Fig. S7), with 747 of them overlapping with the results of LFMM (Fig. 3a). These shared SNPs were associated with 301 genes (gene region and 2.5 kb upstream/downstream). Within the GO terms inthe category of biological processes, these candidate genes were mainly involved with protein localization and protein transport, suggesting the complicated mechanism of local adaptation in *D. involucrata* (Supplementary Table S6).

To evaluate the adaptive potential arising from introgression, we evaluated the introgressive signals and ratios of the two admixture groups. We found 14 windows in the Wmix group and 18 windows in the Emix group with positive *D* values and high introgressive ratios (*f-dM* > 0.15). In these introgressive windows, most alleles were neutral; less than 4% of SNPs were identified as environment-associated outliers in the Wmix (66/1989) and Emix (77/2189) groups (Fig. 3b). These SNPs could participate in the local adaptation of admixed populations. For example, we found a variant located at *Dinv38473* (chr7: 38379580) which encodes a two-component response regulator-like APRR1 orthologous to that in *Nyssa sinensis*^[46]^ (Fig. 3c). APRR1 plays a core role in the circadian clock in plants^[47]^. These results provide evidence of adaptive introgression in *D. involucrata*, suggesting increased adaptive potential as a result of genetic admixture.

### Prediction of genomic vulnerability

**Figure 4:**
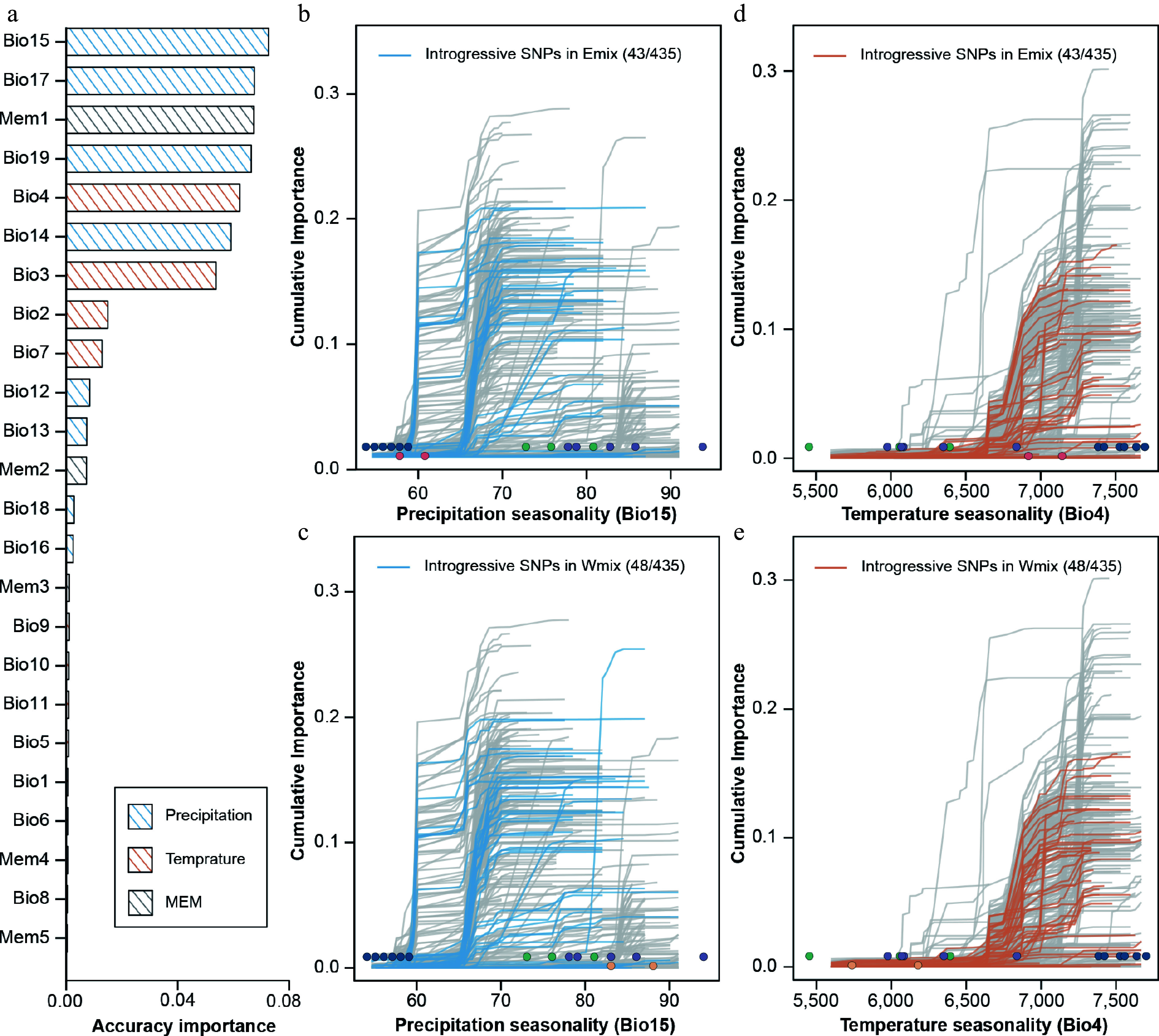
Accuracy importance of all predictors and cumulative importance for two variables in the gradient forest model. (a) Importance ranking of all environmental and spatial variables, showing the allelic turnover functions of 435 climate-associated SNPs relative to two high-ranking bioclimatic variables, i.e., seasonality of precipitation (BIO15) and seasonality of temperature (BIO4). Thin lines show each of the 435 candidate SNPs. For BIO15, the introgressive SNPs identified in the Emix (b) and Wmix (c) groups are highlighted in blue. For BIO4, the introgressive SNPs identified in the Emix (d) and Wmix (e) groups are highlighted in orange. Dots represent the environmental variables of 18 sampling sites, with the colors corresponding to the population structure identified in Figure 1.

GF analysis was applied to model the turnover of adaptive alleles and environmental gradients. We used 19 climatic variables and 5 MEM variables as descriptors of the spatial variation. Here, 58.2% (435/747) of environment-associated SNPs showed a positive *R*^2^ in the model. We found that the seasonality of precipitation (BIO15) was the strongest predictor of the GF model (Fig. 4a). In the turnover function of annual precipitation, the allele frequency sharply changed when annual precipitation reached 60–70 mm (Fig. 4b). In 435 SNPs in GF models, there were 75 introgressive alleles in Wmix and 68 introgressive alleles in Emix. We also mapped the introgressive alleles for the turnover functions of two important variables (BIO15, BIO4). For the Emix group, 43 introgressive alleles showed positive *R*^2^ (Fig. 4b, d). For the Wmix group, 48 introgressive alleles showed a positive *R*^2^ (Fig. 4c, e). However, these introgressive alleles showed low or moderate cumulative importance in the prediction model, suggesting that they are less helpful in responding to future climate change.

**Figure 5:**
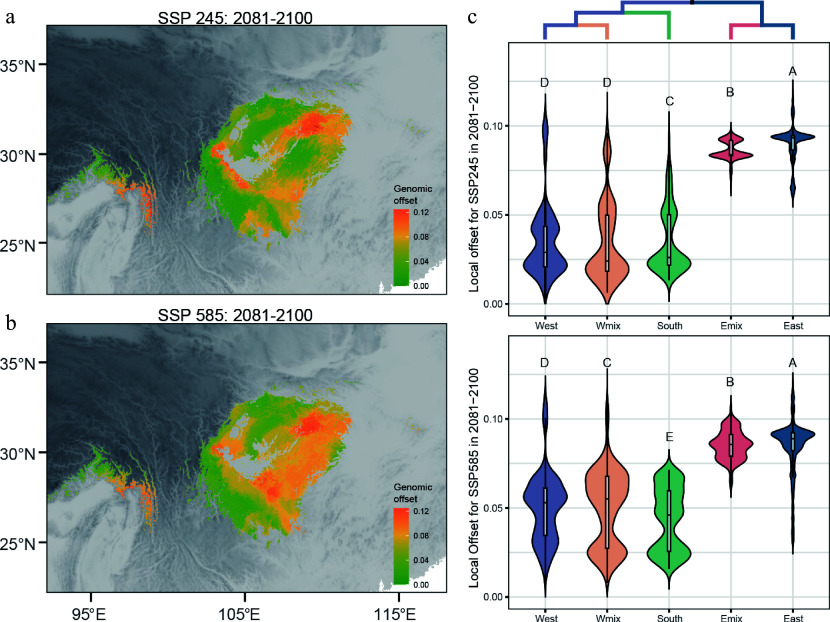
Genomic offset based on GEA outliers for 2081–2100. Local offsets across the range of *D. involucrata* for SSP245 (a) and SSP585 (b). Violin plot of local offsets in three lineages and two admixed groups for SSP245 (c) and SSP585 (d). The phylogenic tree indicates the relationship of the five genetic groups.

On the basis of the GF model described above, we quantified the *in situ* maladaptation of *D. involucrata* populations. In 12 future climatic scenarios, the predicted local offsets showed a similar pattern; populations in the eastern range had a higher risk of maladaptation in the future, covering the eastern lineage and the Emix group (Fig. 5a, c, Supplementary Fig. S8). The wo admixed populations shared similar genetic offsets with their parents' lineages. Under the SSP245 scenario, the genetic offset of the Wmix group did not differ significantly from that of the western lineage (*p =* 0.075), and both exhibited relatively low maladaptive risk. In contrast, the Emix group exhibited significant lower offsets than the eastern lineage (*p* < 2.2 × 10^–16^); however, the average offset in the Emix group (mean local offset: 0.086) was still 96% of that in the eastern lineages (mean local offset: 0.090) (Fig. 5b). Under the SSP585 scenario, the Wmix group exhibited significantly higher genetic offsets than the western lineage (*p* = 1.6 × 10^–6^). The Emix group also showed reduced offsets compared with the eastern lineage (*p* = 2.1 × 10^–5^) (Fig. 5d). These results suggested a limited role of genetic admixture in reducing the maladaptive risk of *D. involucrata*.

**Figure 6:**
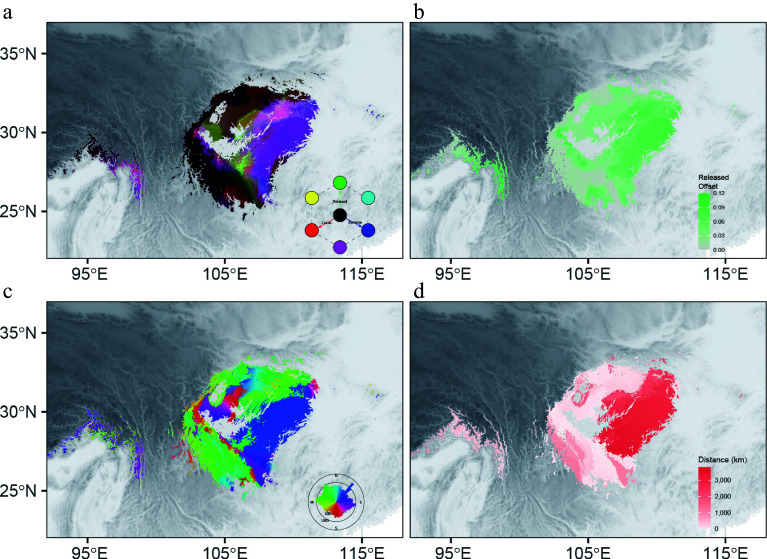
Integrated offset, migration distance, and initial bearing for SSP245 in 2081–2100. (a) Mapping of local offsets (red), forward offsets (green), and reverse offsets (blue). (b) The difference between local offsets and forward offsets. The migration distance to (c) and initial bearing (d) of the locations that minimize forward offset.

Assisted migration to a suitable future habitat is a direct way to minimize the local extinction risk. After considering migration alongside genomic offset, we assessed the forward and reverse offset under SSP245 and SSP585 in 2081–2100. We found that cells with a high local offset exhibited a low forward offset (Fig. 6a, Supplementary Figs S9 and S10) under both the SSP245 and SSP585 scenarios. This suggests that assisted migration would be an effective measure to decrease the maladaptation risk for the eastern population, especially the eastern proportion of the range (Fig. 6b). For populations of the eastern lineage, minimizing future maladaptation implies a necessary displacement of roughly 3000 km to the northeast (Fig. 6c, d). After restricting the maximum allowable migration distance (50, 100, 250, 500 km), we found a lowered forward offset as the migration distances increased (Supplementary Fig. S11). The decline in forward offset between 0 and 250 km was 64.9% across all cells, whereas the average forward offset decreased by only 8.6% when the migration distance expanded from 250 km to unlimited. This result suggested an efficient migration distance of 250 km, which could reach 88.5% of the decline in offset compared with the global minimum offset.

## Discussion

Adopting a dense sampling scheme, we used RAD-seq to confirm a population structure consistent with that revealed in a previous study based on whole-genome resequencing data^[22]^. We reconstructed the demographic history of two admixed population groups. Our results indicated that both the Wmix and Emix groups arose from gene flow at ca. 0.17 Mya. These admixture events coincided with the timing of the Guxiang glaciation (ca. 0.30–0.13 Mya)^[48]^. Additionally, relatively high levels of constant gene flow between the Emix group and the southern lineage were inferred (1.1 × 10^−5^), indicating that significant genetic exchange continues to occur in the dove tree (Fig 2c). Evidence of glacial-period population admixture has also been found in other relict plant species in warm-temperate deciduous forests^[49,50]^. Moreover, genetic admixture contributes to adaptive potential by increasing genetic variation along environmental gradients^[51]^ and also provides important sources of genetic materials for natural selection to act on^[17]^.

Adaptive introgression could partly mitigate genomic vulnerability in the dove tree. The Wmix group and the western lineage exhibited similar offsets, whereas the Emix group exhibited significant lower vulnerability under future climate conditions. However, the reduction was modest (Fig. 5c,d). This subtle effect of adaptive introgression could be explained by the modest effect size of the introgressed alleles and the absence of key adaptive variants. This is consistent with the polygenic nature of many adaptive traits, where candidate loci are often expected to have only small effects on phenotype^[52]^; however, the effect of adaptive variants on traits need empirical validation. The density of our genetic markers also limited the power to detect crucial adaptive alleles for reducing genomic vulnerability. Nevertheless, our results still offer great insight into the effect of adaptive introgression on genomic vulnerability. Further investigation is needed to fully understand the potential evolutionary consequences of introgression for climate change adaptation in relic species.

Genomic vulnerability has been predicted in many species, including several relict plants, such as *Euptelea* spp.^[53]^, *Pterocarya macroptera*^[54]^, and *Taiwania cryptomerioides*^[55]^. These results highlighted that marginal populations located at the edges of the environmental limits are at a higher extinction risk. Consistent with these findings, our study revealed that eastern populations experiencing low precipitation exhibited greater genomic vulnerability, suggesting an increased extinction risk and reduced fitness under climate change. Genetic diversity is a key conservation criterion^[56]^. Here, we found that the populations with the highest diversity may also face the greatest risk of maladaptation. Although these populations have a strong capacity for adaptation, keeping pace with rapid climate change is still challenging for long-lived trees. Our results heighten the conservation priority of the eastern population of *D. involucrata* to preserve its substantial genetic diversity. As genetically distinct lineages show divergent responses to climate change, we highlighted the application of evolutionary significant units (ESUs)^[57]^. According these results, *D. involucrata* falls into five demographically independent population groups. Overall, integrating the definition of ESUs with the assessment of genomic vulnerability provides a framework for prioritizing conservation under future climate scenarios^[58]^.

Plant conservation can be achieved through *in situ* or *ex situ* management^[59]^, whereby genomic vulnerability assessments help us to determine how to protect each ESU by identifying the optimal migration destination. Showing a low level of genomic vulnerability, the western lineage and the Wmix group of *D. involucrata* are suitable candidates for *in situ* conservation. On the contrary, *ex situ* conservation (e.g., transplantation and breeding in botanical gardens and arboreta) may be more practical for populations with high local offsets^[60]^. To reduce the maladaptive risk and avoid strong selection pressures during *ex situ* conservation practices, we predicted the genomic response following introduction to the optimal planting sites by calculating the forward genomic offsets (Supplementary Fig. S6)^[45]^. Encouragingly, most populations exhibited low forward genomic offsets, indicating that they could escape from the extinction risk by artificial migration.

## Conclusions

In summary, we collected and analyzed genetic data from 196 *D. involucrata* individuals to investigate the role of intraspecific introgression in maladaptive risk. After the population structure was confirmed and admixed populations had been identified, we selected candidate SNPs as response variables to predict the genomic vulnerability for each population. Both admixed population groups of *D. involucrata* have introduced some adaptive alleles from the southern lineage, but only the high-risk group exhibited a modest but significant reduction in genomic vulnerability. Finally, we modeled the effects of assisted migration and formulated population-level conservation guidelines. Overall, this study provides a comprehensive genome-wide view of maladaptation risk in *D. involucrata* by integrating multiple lines of evidence from local adaptation, migration, and demographic history. Such information is crucial for both the scientific community and policymakers to develop climate-resilient conservation strategies for relict plants.

## SUPPLEMENTARY DATA

Supplementary data to this article can be found online.

## Data Availability

All data generated in this study have been deposited in the National Genomics Data Center (NGDC, https://ngdc.cncb.ac.cn) under accession number PRJCA032460.
