# Supplementary material for: Genomic vulnerability assessment reveals the potential benefits of adaptive introgression by mitigating the maladaptive risk of admixed populations
Source: For Res (Fayettev). 2025 Nov 19;5:e026. doi: 10.48130/forres-0025-0026 (PMC12648016; doi:10.48130/forres-0025-0026)
Supplement: Supplementary file 1 — Supplementary data to this article can be found online. [file FR-2025-5-0026-Supplementary.zip › 10.48130_forres-0025-0026-Suppl-FigureS8.pdf]

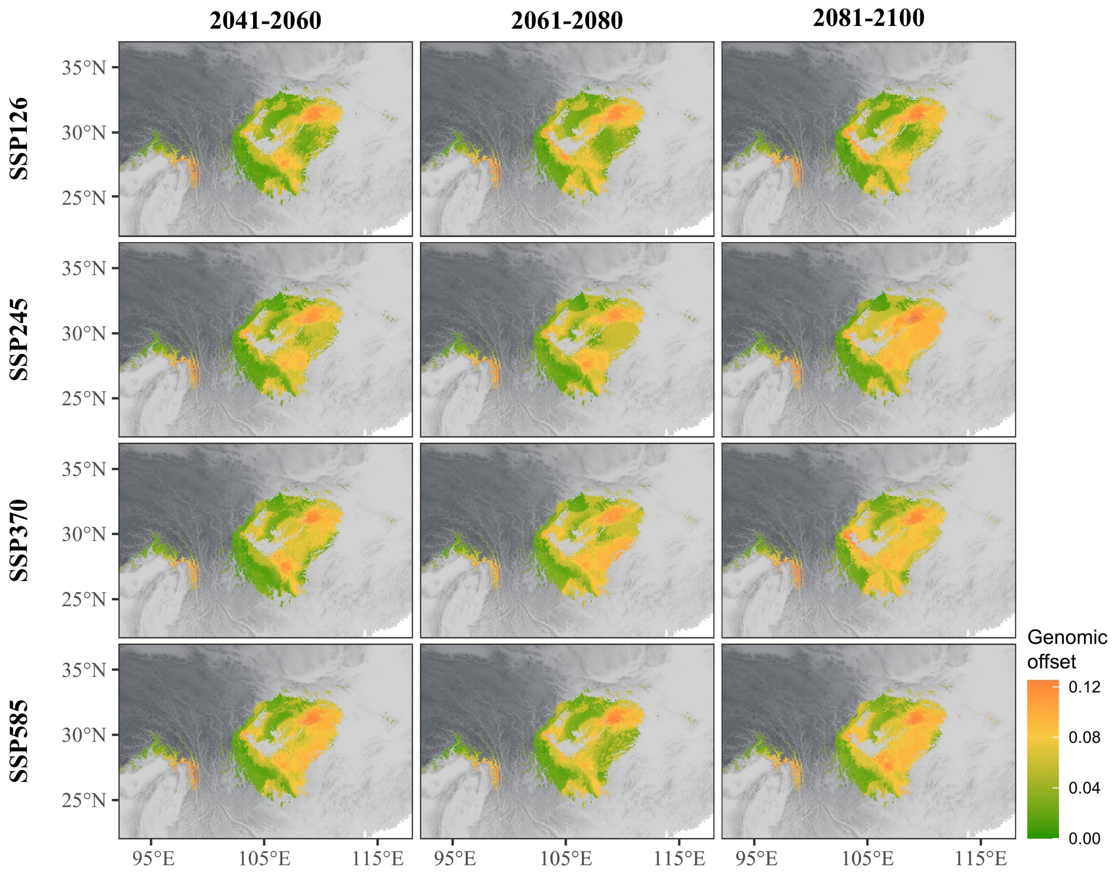

Figure S8 Local genetic offset from a Gradient Forest model for four SSP scenarios and for 2041-2060, 2061-2080, 2081-2100. A color gradient ranging from green (low values) to orange (high values) visually represents the magnitude of offset values.
