# Supplementary material for: Genomic vulnerability assessment reveals the potential benefits of adaptive introgression by mitigating the maladaptive risk of admixed populations
Source: For Res (Fayettev). 2025 Nov 19;5:e026. doi: 10.48130/forres-0025-0026 (PMC12648016; doi:10.48130/forres-0025-0026)
Supplement: Supplementary file 1 — Supplementary data to this article can be found online. [file FR-2025-5-0026-Supplementary.zip › 10.48130_forres-0025-0026-Suppl-FigureS9.pdf]

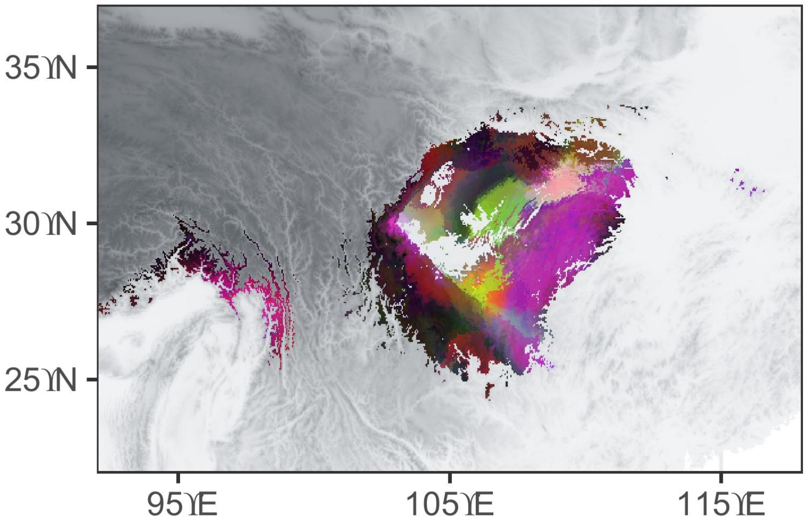

Figure S9 The RGB map of local (red), forward (green) and reverse (blue) genomic offsets. The offset values were summarized from the GF model throughout the distribution range of *Davidia involucrata* for the year 2081-2100 and the SSP585 scenario.
