# Supplementary material for: Genomic vulnerability assessment reveals the potential benefits of adaptive introgression by mitigating the maladaptive risk of admixed populations
Source: For Res (Fayettev). 2025 Nov 19;5:e026. doi: 10.48130/forres-0025-0026 (PMC12648016; doi:10.48130/forres-0025-0026)
Supplement: Supplementary file 1 — Supplementary data to this article can be found online. [file FR-2025-5-0026-Supplementary.zip › 10.48130_forres-0025-0026-Suppl-TableS1.pdf]

Table S1 Sampling information and a summary of genetic diversity of studied *D. involucrata* populations.

Pop.ID	Longitude/°E	Latitude/°N	n	π (×10^{-4})	Ho/He (×10^{-2})
**West**			**47**	**1.40**	**0.32/5.78**
SCBC	104.32	31.86	5	0.92	0.34/6.09
SCWC	103.27	30.88	8	1.11	0.57/4.85
SCBX	102.64	30.41	11	1.04	0.36/4.92
SCTQ	102.43	30.17	11	1.08	0.43/4.91
SCYJ	102.89	29.63	14	1.04	0.30/4.59
**Wmix**			28	**1.51**	**0.45/6.10**
SCEM	103.35	29.56	13	1.16	0.32/4.42
SCLB	103.64	28.38	15	1.41	0.58/5.53
**South**			22	**3.42**	**10.3/13.9**
SCJL	104.76	27.89	12	1.28	0.28/4.42
GZYS	104.77	26.5	5	3.43	16.0/12.9
GZZJ	105.55	26.57	5	3.64	17.8/13.7
**Emix**			26	**1.97**	**0.64/7.94**
GZKK	107.17	28.23	15	1.74	0.47/6.99
CQJF	107.05	28.87	11	1.75	0.88/7.06
**East**			**69**	**2.11**	**0.49/8.69**
GZFJ	108.77	27.84	13	1.66	0.66/7.23
HNTP	110.09	29.78	14	1.70	0.51/7.72
HNZJJ	110.41	29.33	6	0.96	0.30/4.06
HBXD	109.11	30.09	16	1.66	0.47/7.36
HBDLL	110.93	31.06	7	1.31	0.53/5.56
HBSNJ	110.39	31.47	15	1.51	0.43/6.43
