# Supplementary material for: Genomic vulnerability assessment reveals the potential benefits of adaptive introgression by mitigating the maladaptive risk of admixed populations
Source: For Res (Fayettev). 2025 Nov 19;5:e026. doi: 10.48130/forres-0025-0026 (PMC12648016; doi:10.48130/forres-0025-0026)
Supplement: Supplementary file 1 — Supplementary data to this article can be found online. [file FR-2025-5-0026-Supplementary.zip › 10.48130_forres-0025-0026-Suppl-TableS2.pdf]

Table S2 Overview of sample information and sequencing statistics of *D. involucrata*.

No.	Sample ID	Population ID	cleandata size (Gb)	porperly paired rate
1	SCBX01	SCBX	2.8	88.55%
2	SCBX02	SCBX	2.0	81.08%
3	SCBX03	SCBX	1.8	85.88%
4	SCBX04	SCBX	1.0	82.06%
5	SCBX05	SCBX	1.6	80.98%
6	SCBX06	SCBX	3.0	88.06%
7	SCBX07	SCBX	1.9	78.02%
8	SCBX08	SCBX	2.1	89.58%
9	SCBX09	SCBX	1.2	84.99%
10	SCBX10	SCBX	1.4	86.48%
11	SCBX11	SCBX	2.7	87.03%
12	SCBC01	SCBC	1.1	86.98%
13	SCBC02	SCBC	2.5	89.62%
14	SCBC03	SCBC	1.6	87.52%
15	SCBC04	SCBC	1.3	86.88%
16	SCBC05	SCBC	2.2	88.37%
17	HBDLL01	HBDLL	1.7	83.91%
18	HBDLL02	HBDLL	6.7	74.59%
19	HBDLL03	HBDLL	1.0	84.63%
20	HBDLL04	HBDLL	1.5	84.19%
21	HBDLL05	HBDLL	1.4	85.47%
22	HBDLL06	HBDLL	2.5	84.90%
23	HBDLL07	HBDLL	1.0	85.37%
24	SCEM01	SCEM	2.5	89.09%
25	SCEM02	SCEM	2.8	90.07%
26	SCEM03	SCEM	1.0	88.80%
27	SCEM04	SCEM	1.6	89.30%
28	SCEM05	SCEM	1.4	88.34%
29	SCEM06	SCEM	1.5	89.64%
30	SCEM07	SCEM	2.2	88.96%
31	SCEM08	SCEM	1.2	87.69%
32	SCEM09	SCEM	1.4	87.33%
33	SCEM10	SCEM	2.9	87.33%
34	SCEM11	SCEM	1.4	88.44%
35	SCEM12	SCEM	2.9	87.77%
36	SCEM13	SCEM	1.8	90.13%
37	GZFJ01	GZFJ	4.8	84.17%
38	GZFJ02	GZFJ	6.3	80.42%
39	GZFJ03	GZFJ	5.8	78.96%
40	GZFJ04	GZFJ	2.2	84.33%
41	GZFJ05	GZFJ	3.9	82.71%
42	GZFJ06	GZFJ	1.9	82.21%

No.	Sample ID	Population ID	cleandata size (Gb)	porperly paired rate
43	GZFJ07	GZFJ	3.0	84.22%
44	GZFJ08	GZFJ	2.2	84.51%
45	GZFJ09	GZFJ	2.5	82.32%
46	GZFJ10	GZFJ	10.0	74.65%
47	GZFJ11	GZFJ	3.8	86.27%
48	GZFJ12	GZFJ	3.2	86.26%
49	GZFJ13	GZFJ	3.8	85.44%
50	CQJF01	CQJF	3.4	87.60%
51	CQJF02	CQJF	2.2	87.64%
52	CQJF03	CQJF	2.3	87.96%
53	CQJF04	CQJF	0.4	88.59%
54	CQJF05	CQJF	0.5	90.09%
55	CQJF06	CQJF	3.2	89.66%
56	CQJF07	CQJF	4.3	89.50%
57	CQJF08	CQJF	0.2	89.69%
58	CQJF09	CQJF	5.6	84.60%
59	CQJF10	CQJF	2.0	89.47%
60	CQJF11	CQJF	1.7	89.19%
61	SCJL01	SCJL	2.2	86.33%
62	SCJL02	SCJL	2.3	88.28%
63	SCJL03	SCJL	1.9	87.15%
64	SCJL04	SCJL	0.9	88.78%
65	SCJL05	SCJL	1.9	87.05%
66	SCJL06	SCJL	2.0	78.78%
67	SCJL07	SCJL	3.2	88.31%
68	SCJL08	SCJL	2.6	86.45%
69	SCJL09	SCJL	2.3	83.40%
70	SCJL10	SCJL	1.8	83.92%
71	SCJL11	SCJL	3.6	82.35%
72	SCJL12	SCJL	4.8	87.27%
73	GZKK01	GZKK	1.7	79.04%
74	GZKK02	GZKK	3.1	88.14%
75	GZKK03	GZKK	3.1	88.22%
76	GZKK04	GZKK	2.2	87.23%
77	GZKK05	GZKK	1.0	85.19%
78	GZKK06	GZKK	3.7	84.04%
79	GZKK07	GZKK	1.0	88.03%
80	GZKK08	GZKK	1.5	86.39%
81	GZKK09	GZKK	3.2	85.07%
82	GZKK10	GZKK	1.4	79.74%
83	GZKK11	GZKK	5.1	80.61%
84	GZKK12	GZKK	0.2	88.03%
85	GZKK13	GZKK	2.2	86.12%

No.	Sample ID	Population ID	cleandata size (Gb)	porperly paired rate
86	GZKK14	GZKK	1.1	85.84%
87	GZKK15	GZKK	1.5	86.43%
88	SCLB01	SCLB	3.5	89.41%
89	SCLB02	SCLB	6.1	90.21%
90	SCLB03	SCLB	5.0	87.27%
91	SCLB04	SCLB	3.4	90.41%
92	SCLB05	SCLB	3.2	87.65%
93	SCLB06	SCLB	2.5	93.77%
94	SCLB07	SCLB	4.2	86.83%
95	SCLB08	SCLB	2.3	91.86%
96	SCLB09	SCLB	1.9	88.47%
97	SCLB10	SCLB	5.4	92.77%
98	SCLB11	SCLB	2.9	87.98%
99	SCLB12	SCLB	2.5	90.01%
100	SCLB13	SCLB	1.8	86.55%
101	SCLB14	SCLB	1.6	92.08%
102	SCLB15	SCLB	2.1	91.23%
103	HBSN01	HBSN	3.7	77.92%
104	HBSN02	HBSN	3.6	80.65%
105	HBSN03	HBSN	3.3	82.67%
106	HBSN04	HBSN	4.8	76.50%
107	HBSN05	HBSN	4.4	83.41%
108	HBSN06	HBSN	1.7	81.83%
109	HBSN07	HBSN	2.0	79.60%
110	HBSN08	HBSN	1.2	80.78%
111	HBSN09	HBSN	1.6	78.61%
112	HBSN10	HBSN	1.5	81.45%
113	HBSN11	HBSN	1.7	82.88%
114	HBSN12	HBSN	2.6	84.26%
115	HBSN13	HBSN	4.3	81.08%
116	HBSN14	HBSN	2.6	81.59%
117	HBSN15	HBSN	3.1	79.51%
118	HNTP01	HNTP	1.8	80.33%
119	HNTP02	HNTP	3.6	78.79%
120	HNTP03	HNTP	5.9	79.00%
121	HNTP04	HNTP	2.4	84.78%
122	HNTP05	HNTP	2.2	84.85%
123	HNTP06	HNTP	0.6	85.26%
124	HNTP07	HNTP	2.9	82.57%
125	HNTP08	HNTP	2.4	79.49%
126	HNTP09	HNTP	2.2	74.70%
127	HNTP10	HNTP	3.5	73.37%
128	HNTP11	HNTP	4.3	80.63%

No.	Sample ID	Population ID	cleandata size (Gb)	porperly paired rate
129	HNTP12	HNTP	1.9	82.30%
130	HNTP13	HNTP	1.9	75.68%
131	HNTP14	HNTP	2.0	74.69%
132	SCTQ01	SCTQ	2.2	86.33%
133	SCTQ02	SCTQ	1.7	88.89%
134	SCTQ03	SCTQ	2.0	87.66%
135	SCTQ04	SCTQ	3.6	88.96%
136	SCTQ05	SCTQ	1.2	89.46%
137	SCTQ06	SCTQ	2.6	88.12%
138	SCTQ07	SCTQ	2.3	88.92%
139	SCTQ08	SCTQ	2.3	88.65%
140	SCTQ09	SCTQ	3.2	91.30%
141	SCTQ10	SCTQ	3.1	88.98%
142	SCTQ11	SCTQ	3.0	91.34%
143	SCWC01	SCWC	1.6	80.63%
144	SCWC02	SCWC	1.1	76.37%
145	SCWC03	SCWC	1.2	70.47%
146	SCWC04	SCWC	2.1	81.75%
147	SCWC05	SCWC	1.7	89.64%
148	SCWC06	SCWC	3.7	80.84%
149	SCWC07	SCWC	4.8	78.80%
150	SCWC08	SCWC	3.8	88.13%
151	HBXD01	HBXD	3.5	72.02%
152	HBXD02	HBXD	3.4	75.93%
153	HBXD03	HBXD	5.4	87.30%
154	HBXD04	HBXD	2.1	80.60%
155	HBXD05	HBXD	4.2	81.48%
156	HBXD06	HBXD	4.3	79.16%
157	HBXD07	HBXD	2.9	79.33%
158	HBXD08	HBXD	2.9	78.29%
159	HBXD09	HBXD	2.9	73.68%
160	HBXD10	HBXD	7.3	73.18%
161	HBXD11	HBXD	3.0	78.34%
162	HBXD12	HBXD	1.3	83.19%
163	HBXD13	HBXD	2.4	80.07%
164	HBXD14	HBXD	6.1	80.66%
165	HBXD15	HBXD	0.4	80.37%
166	HBXD16	HBXD	2.9	76.62%
167	SCYJ01	SCYJ	2.1	90.50%
168	SCYJ02	SCYJ	2.6	88.39%
169	SCYJ03	SCYJ	1.4	86.79%
170	SCYJ04	SCYJ	2.0	86.54%
171	SCYJ05	SCYJ	2.5	86.96%

No.	Sample ID	Population ID	cleandata size (Gb)	porperly paired rate
172	SCYJ06	SCYJ	2.1	86.29%
173	SCYJ07	SCYJ	1.6	88.74%
174	SCYJ08	SCYJ	1.6	87.41%
175	SCYJ09	SCYJ	2.1	88.64%
176	SCYJ10	SCYJ	1.1	89.71%
177	SCYJ11	SCYJ	2.1	86.17%
178	SCYJ12	SCYJ	1.7	89.03%
179	SCYJ13	SCYJ	2.4	87.87%
180	SCYJ14	SCYJ	4.1	88.45%
181	HNZJJ01	HNZJJ	2.5	82.08%
182	HNZJJ02	HNZJJ	2.4	80.05%
183	HNZJJ03	HNZJJ	2.8	82.15%
184	HNZJJ04	HNZJJ	1.4	84.67%
185	HNZJJ05	HNZJJ	3.9	80.73%
186	HNZJJ06	HNZJJ	4.4	82.25%
