# Supplementary material for: Genomic vulnerability assessment reveals the potential benefits of adaptive introgression by mitigating the maladaptive risk of admixed populations
Source: For Res (Fayettev). 2025 Nov 19;5:e026. doi: 10.48130/forres-0025-0026 (PMC12648016; doi:10.48130/forres-0025-0026)
Supplement: Supplementary file 1 — Supplementary data to this article can be found online. [file FR-2025-5-0026-Supplementary.zip › 10.48130_forres-0025-0026-Suppl-TableS3.pdf]

Table S3 Cross-validation error for *K* from 1 to 10 in ADMIXTURE analysis.

K	CV error
1	0.466
2	0.404
**3**	**0.391**
4	0.395
5	0.394
6	0.401
7	0.413
8	0.424
9	0.427
10	0.450
