# Supplementary material for: Genomic vulnerability assessment reveals the potential benefits of adaptive introgression by mitigating the maladaptive risk of admixed populations
Source: For Res (Fayettev). 2025 Nov 19;5:e026. doi: 10.48130/forres-0025-0026 (PMC12648016; doi:10.48130/forres-0025-0026)
Supplement: Supplementary file 1 — Supplementary data to this article can be found online. [file FR-2025-5-0026-Supplementary.zip › 10.48130_forres-0025-0026-Suppl-TableS4.pdf]

Table S4 Likelihoods of the 8 candidate demographic models using fastsimcoal2.7.

Model	No. parameters	ΔLikelihood	AIC	ΔAIC	Model normalized relative likelihood (w_i)
5Pop_model_1	8	7244	120,571.8	595.5	0
5Pop_model_2	12	7350	120,846.4	870.1	0
5Pop_model_3	14	7034	120,023.1	46.9	0
5Pop_model_4	16	7091	120,171.6	195.4	0
5Pop_model_5	21	7257	120,604.1	627.8	0
5Pop_model_6	18	7061	120,093.5	117.2	0
**5Pop_model_7**	17	7016	119,976.2	0	0
5Pop_model_8	17	7040	120,038.8	62.5	1
