# Supplementary material for: Genomic vulnerability assessment reveals the potential benefits of adaptive introgression by mitigating the maladaptive risk of admixed populations
Source: For Res (Fayettev). 2025 Nov 19;5:e026. doi: 10.48130/forres-0025-0026 (PMC12648016; doi:10.48130/forres-0025-0026)
Supplement: Supplementary file 1 — Supplementary data to this article can be found online. [file FR-2025-5-0026-Supplementary.zip › 10.48130_forres-0025-0026-Suppl-TableS5.pdf]

Table S5 The 95% confidence interval (CI) for all parameters of the best model in fastsimcoal2.7.

Parameter	Lower	Best estimate	Upper
N_{W}	1,011,132	1,135,957	1,331,731
N_{E}	1,019,077	1,128,365	1,315,648
N_{S}	102,686	100,898	104,956
N_{EM}	148,859	149,068	153,979
N_{WM}	210,656	211,235	219,219
T_{DIV}	4,249,170	4,794,260	5,220,430
T_{SOUTH}	193,460	203,680	209,630
T_{ADMIX}	169,740	174,460	179,230
$AncM_{W\&E}$	2.87E-06	3.60E-06	4.60E-06
$AncM_{W\&S}$	1.39E-05	1.40E-05	1.47E-05
$AncM_{S\&E}$	2.54E-06	4.20E-06	2.85E-06
$M_{W\&E}$	2.41E-07	2.52E-07	3.10E-07
$M_{E\&EM}$	1.20E-06	1.50E-06	1.58E-06
$M_{S\&EM}$	1.09E-05	1.10E-05	1.43E-05
$M_{W\&WM}$	1.88E-06	2.05E-06	2.10E-06
$M_{S\&EM}$	1.95E-06	1.20E-06	2.10E-06
$Ratios_{S2EM}$	2.89E-03	3.05E-3	4.66E-3
