# Supplementary material for: Genomic vulnerability assessment reveals the potential benefits of adaptive introgression by mitigating the maladaptive risk of admixed populations
Source: For Res (Fayettev). 2025 Nov 19;5:e026. doi: 10.48130/forres-0025-0026 (PMC12648016; doi:10.48130/forres-0025-0026)
Supplement: Supplementary file 1 — Supplementary data to this article can be found online. [file FR-2025-5-0026-Supplementary.zip › 10.48130_forres-0025-0026-Suppl-TableS6.pdf]

Table S6 Gene Ontology (GO) annotation of top 20 candidate adaptive genes.

GO term	Discription	Annotated	$-\log_{10}(p)$
GO:0072594	establishment of protein localization to organelle	372	2.79
GO:0006886	intracellular protein transport	729	5.47
GO:0033365	protein localization to organelle	483	3.62
GO:0046907	intracellular transport	1069	8.01
GO:0051649	establishment of localization in cell	1112	8.34
GO:0015031	protein transport	1252	9.39
GO:0045184	establishment of protein localization	1276	9.57
GO:0006605	protein targeting	263	1.97
GO:0008104	protein localization	1415	10.61
GO:0070727	cellular macromolecule localization	1415	10.61
GO:0071705	nitrogen compound transport	1473	11.04
GO:0034504	protein localization to nucleus	114	0.85
GO:0071702	organic substance transport	1686	12.64
GO:0006810	transport	2628	19.7
GO:0033036	macromolecule localization	1627	12.2
GO:0051234	establishment of localization	2683	20.11
GO:0007005	mitochondrion organization	249	1.87
GO:0051641	cellular localization	1752	13.13
GO:0034058	endosomal vesicle fusion	11	0.08
GO:0007034	vacuolar transport	134	1
