# Supplementary figures and images for: Genomic vulnerability assessment reveals the potential benefits of adaptive introgression by mitigating the maladaptive risk of admixed populations
Source: For Res (Fayettev). 2025 Nov 19;5:e026. doi: 10.48130/forres-0025-0026 (PMC12648016; doi:10.48130/forres-0025-0026)

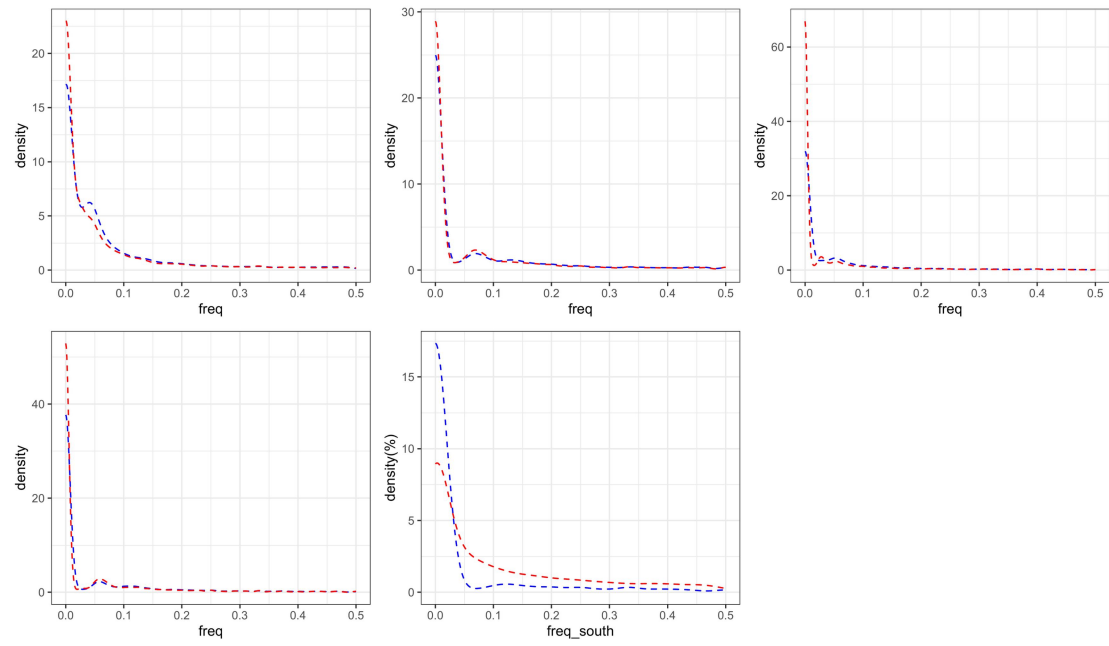

**Figure S1** The SNPs frequency for each lineage of *D. involucrata* with different datasets.

Supplement: Supplementary file 1 — Supplementary data to this article can be found online. [file FR-2025-5-0026-Supplementary.zip › 10.48130_forres-0025-0026-Suppl-FigureS1.pdf]

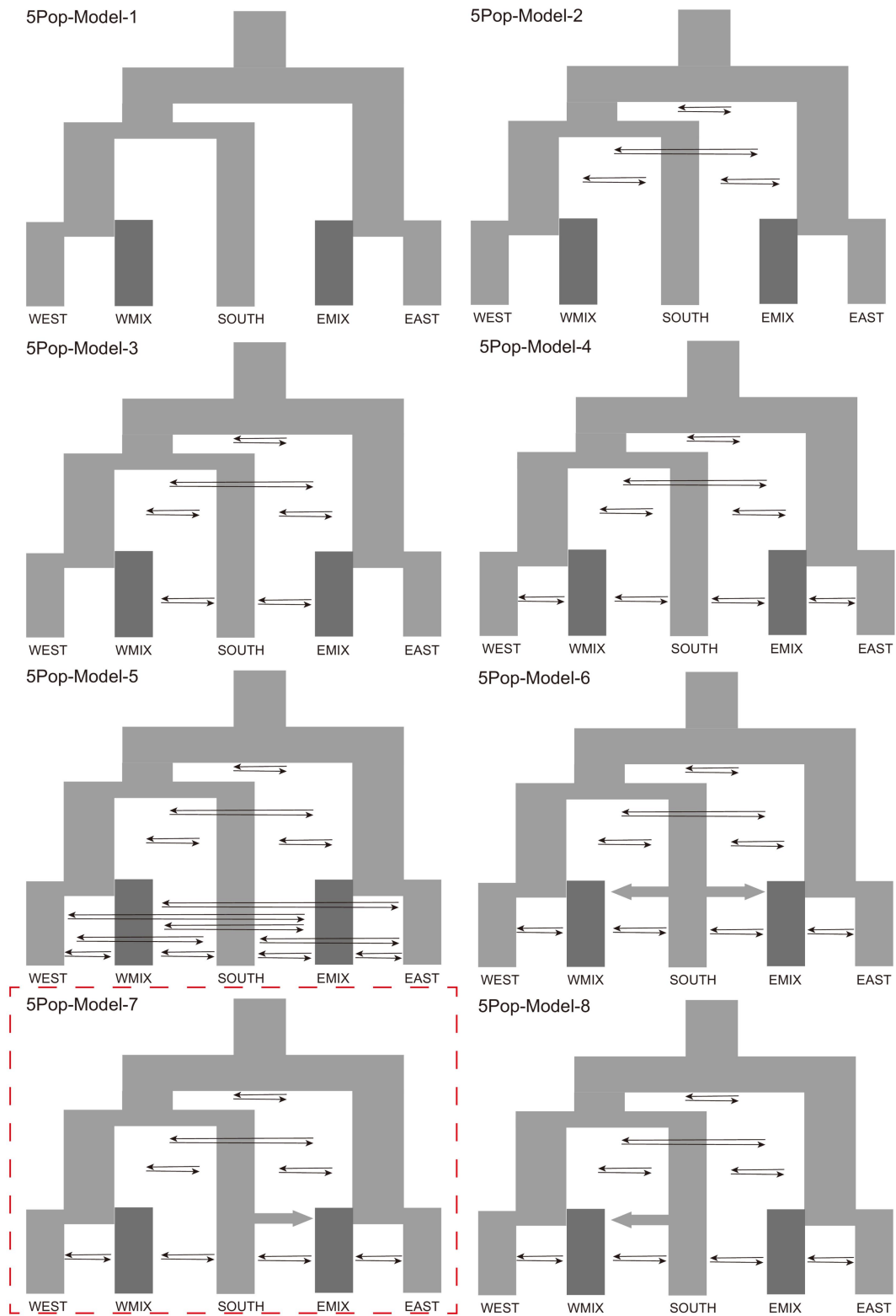

**Figure S2** Diagram of 12 candidate demographic models of five clusters using fastsimcoal2.7.

Supplement: Supplementary file 1 — Supplementary data to this article can be found online. [file FR-2025-5-0026-Supplementary.zip › 10.48130_forres-0025-0026-Suppl-FigureS2.pdf]

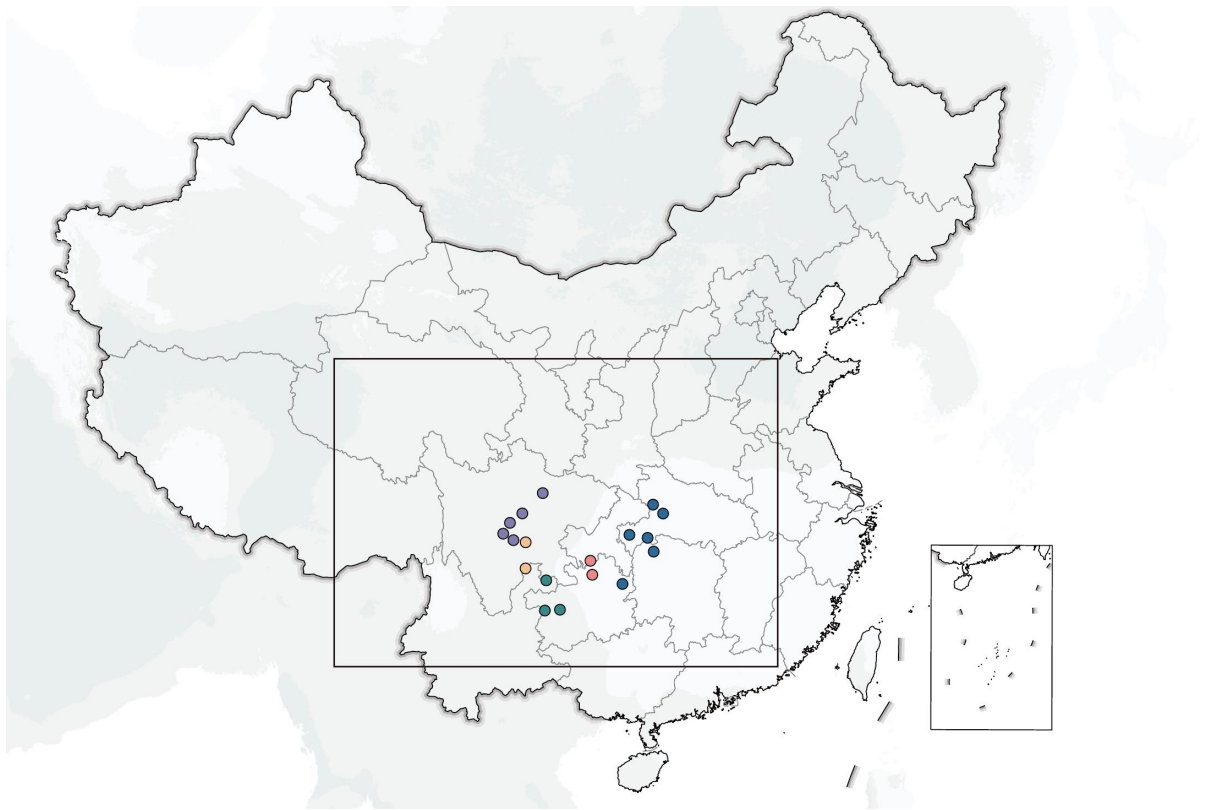

**Figure S4** Map showing the study area of genetic results in Fig. 5 & 6.

Supplement: Supplementary file 1 — Supplementary data to this article can be found online. [file FR-2025-5-0026-Supplementary.zip › 10.48130_forres-0025-0026-Suppl-FigureS4.pdf]

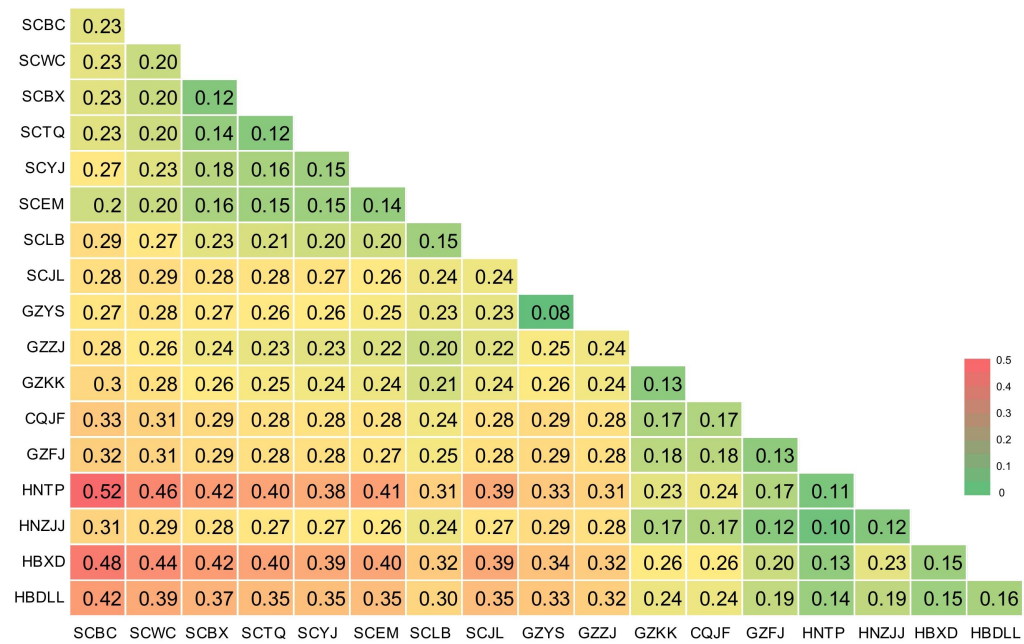

**Figure S5** Pairwise fixation index ( $F_{ST}$ ) among populations in *D. involucrata*.

Supplement: Supplementary file 1 — Supplementary data to this article can be found online. [file FR-2025-5-0026-Supplementary.zip › 10.48130_forres-0025-0026-Suppl-FigureS5.pdf]

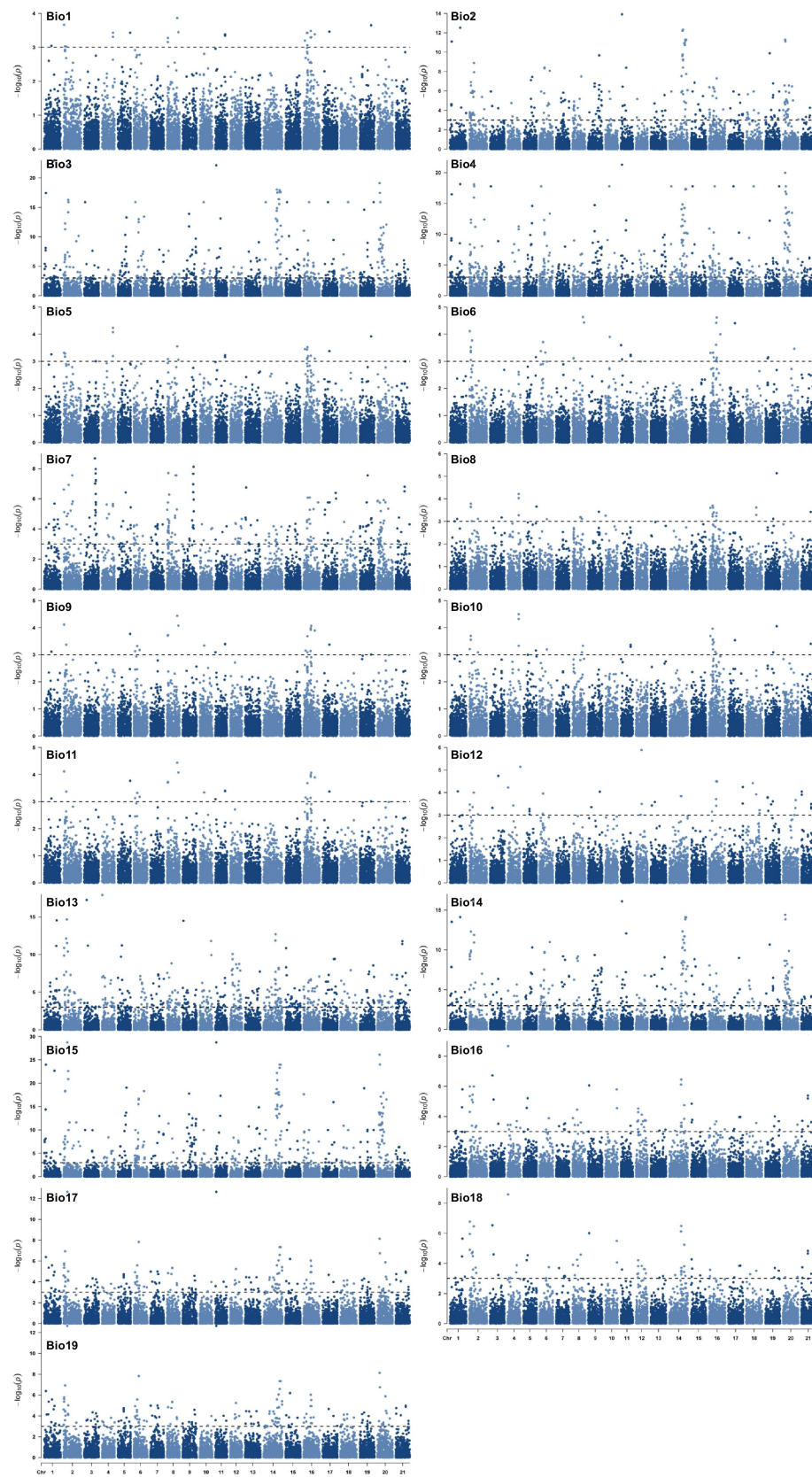

**Figure S6** Manhattan plot of 16569 SNPs with 19 climatic variables in LFMM analysis.

Supplement: Supplementary file 1 — Supplementary data to this article can be found online. [file FR-2025-5-0026-Supplementary.zip › 10.48130_forres-0025-0026-Suppl-FigureS6.pdf]
